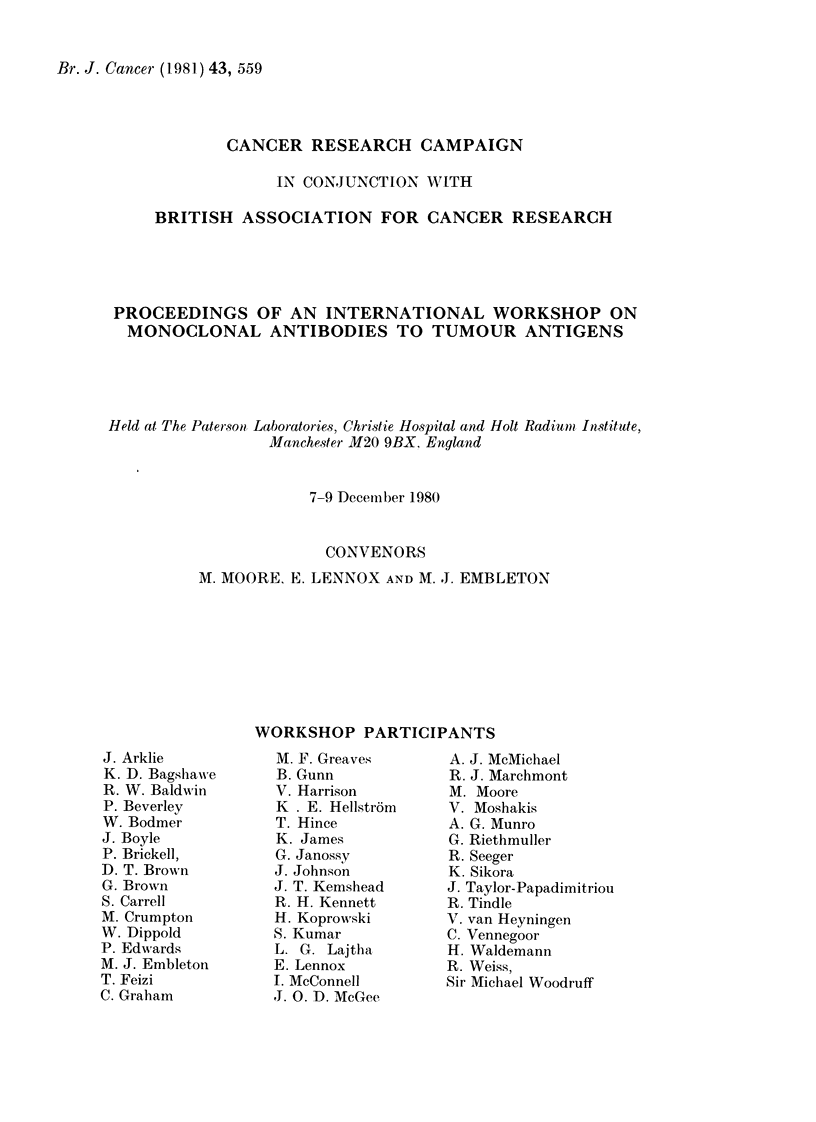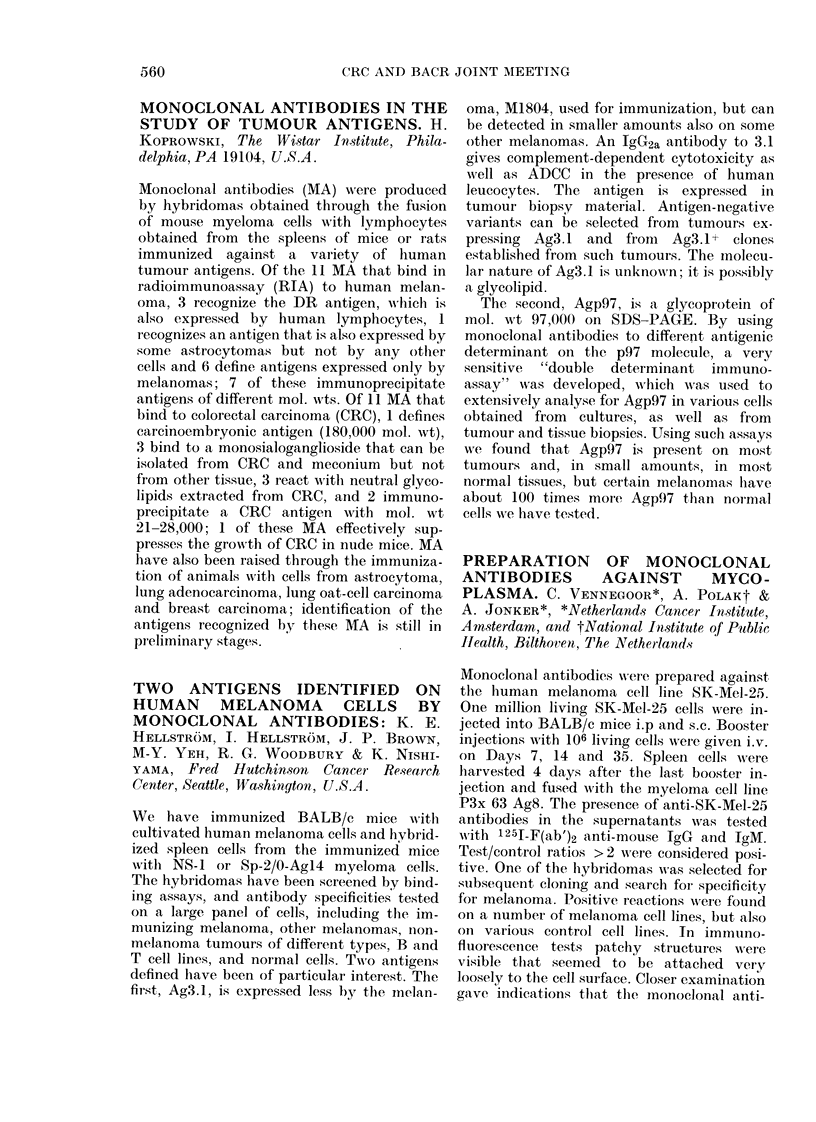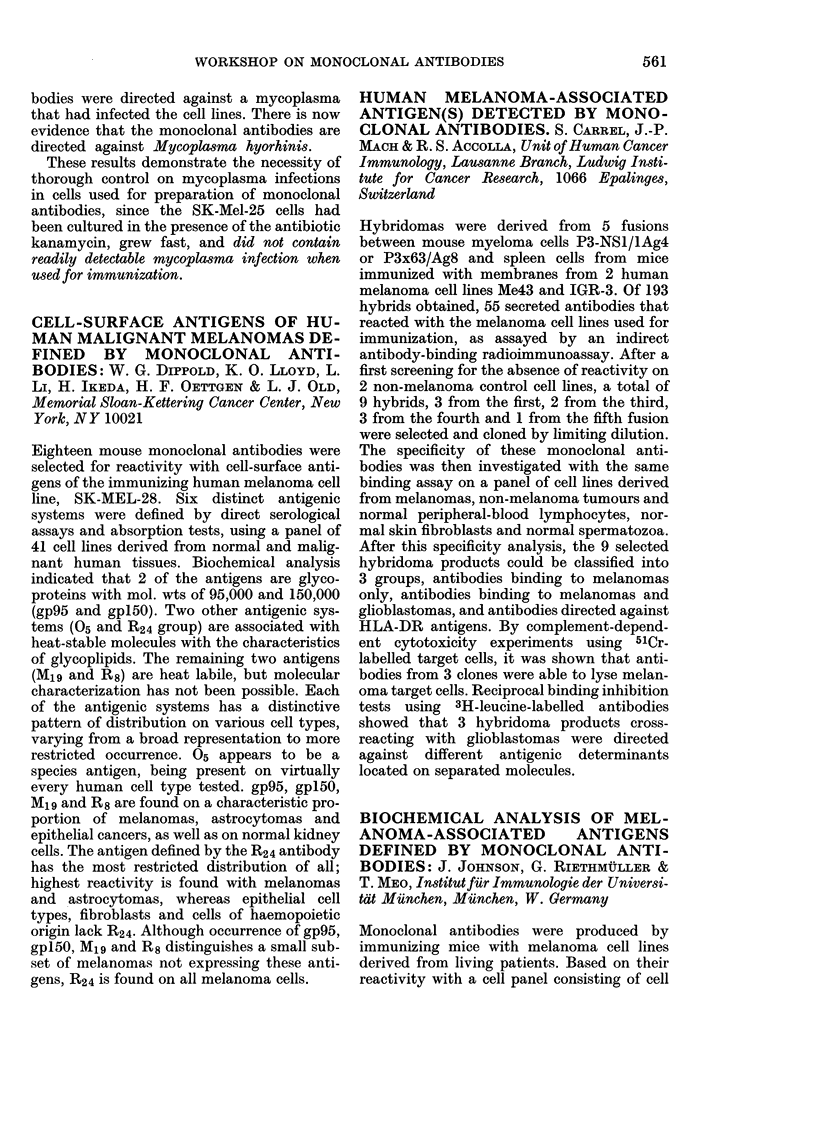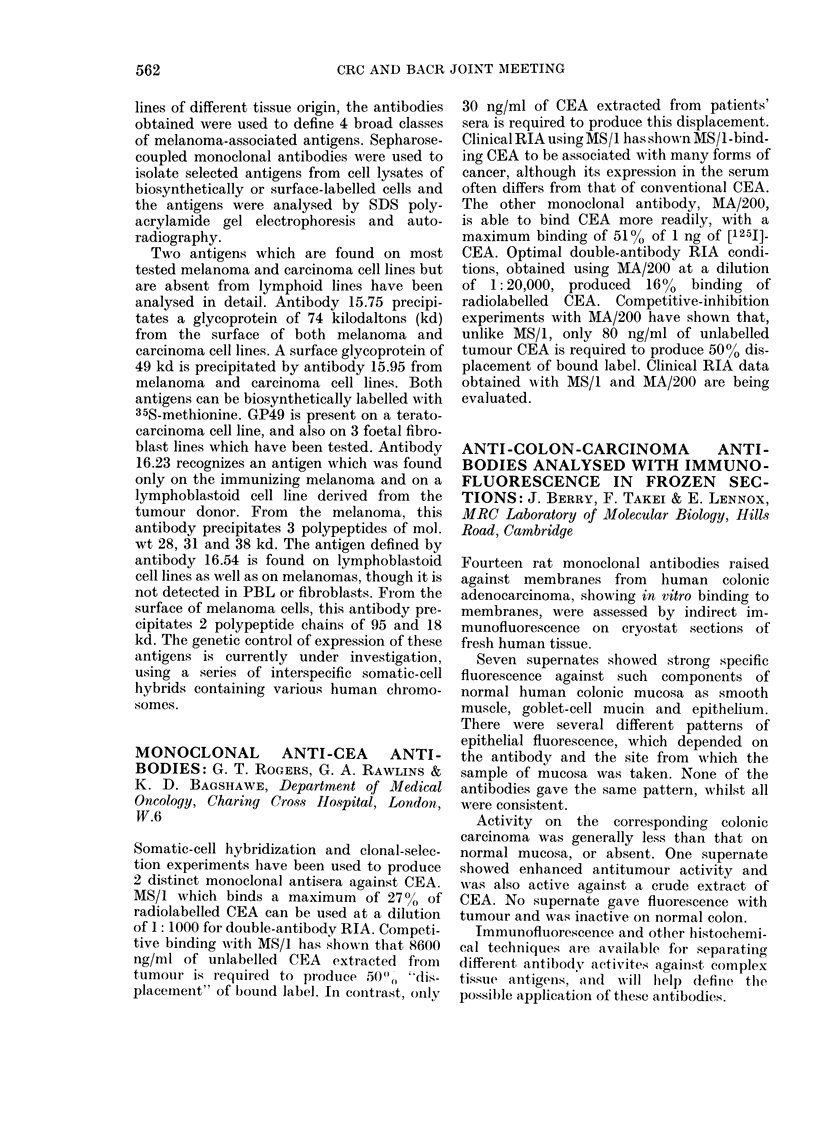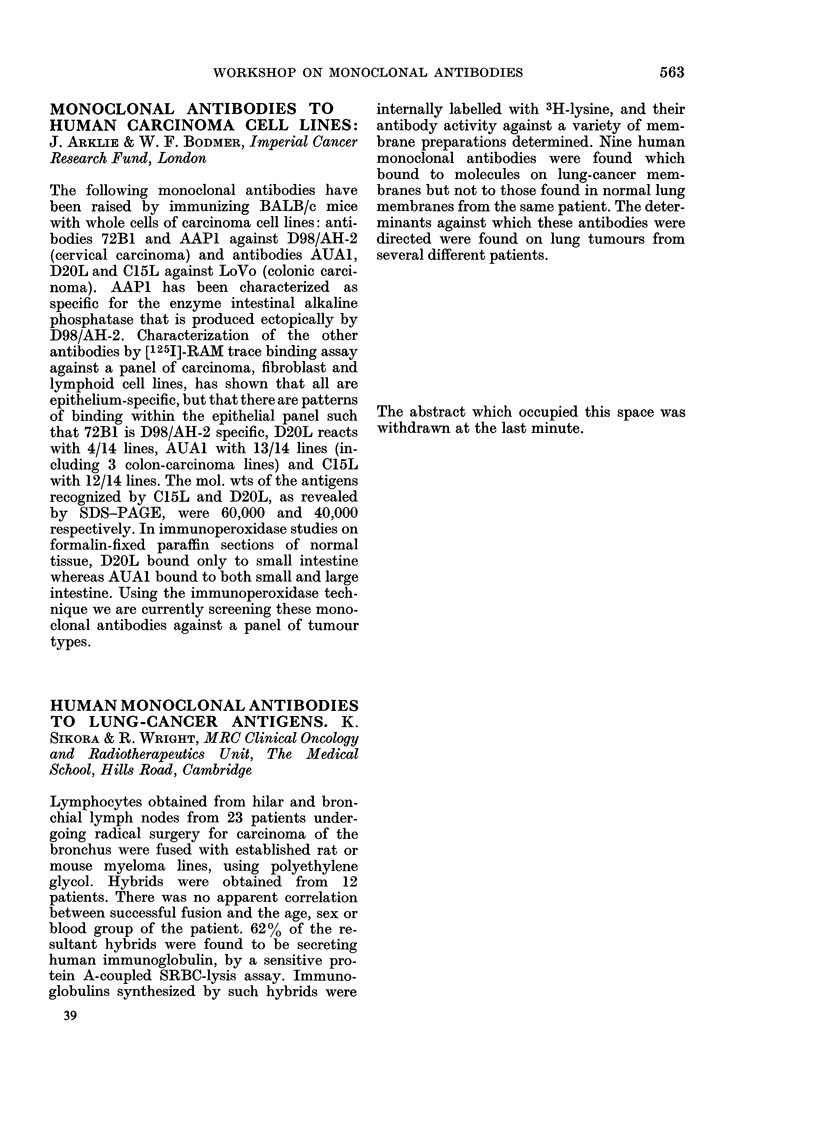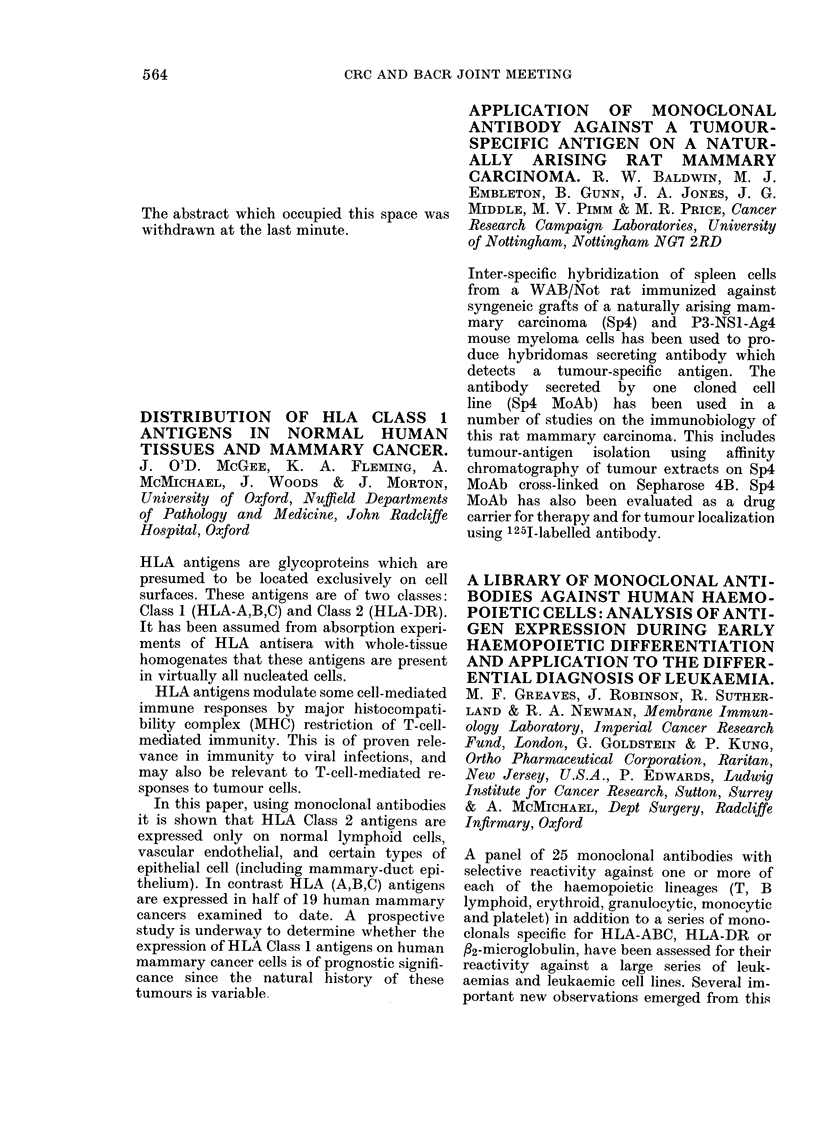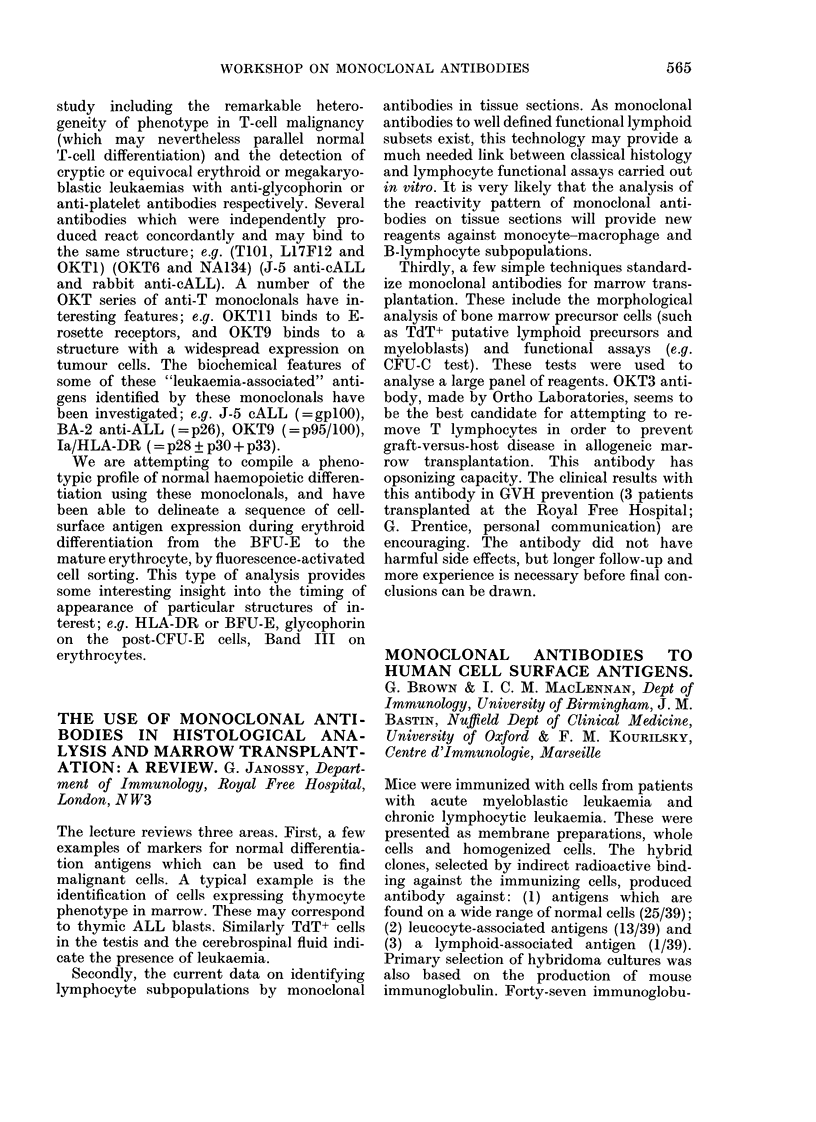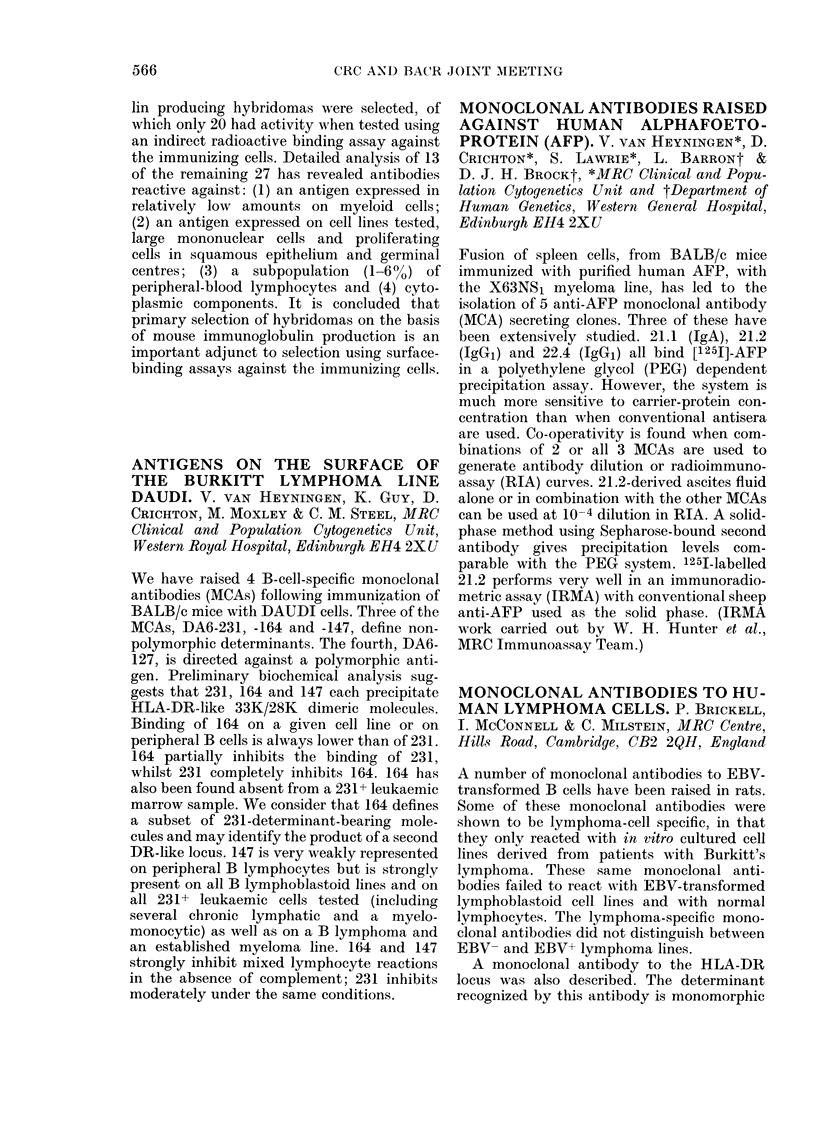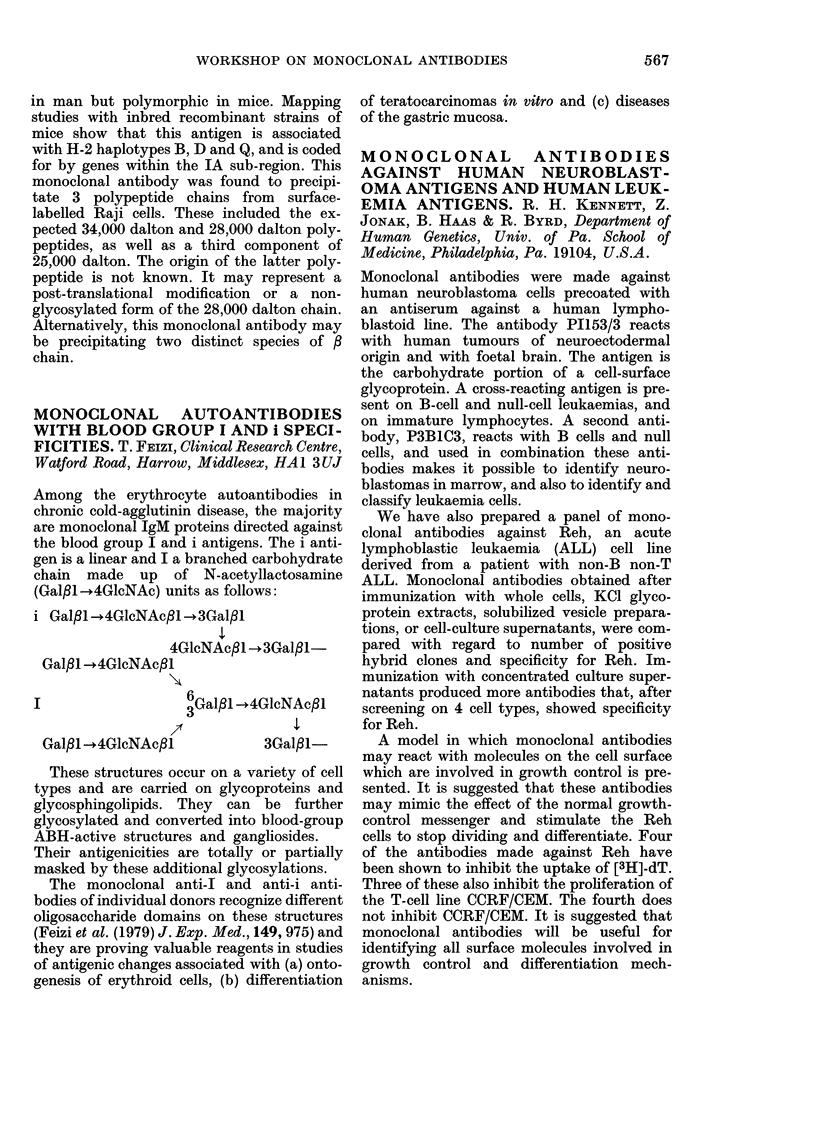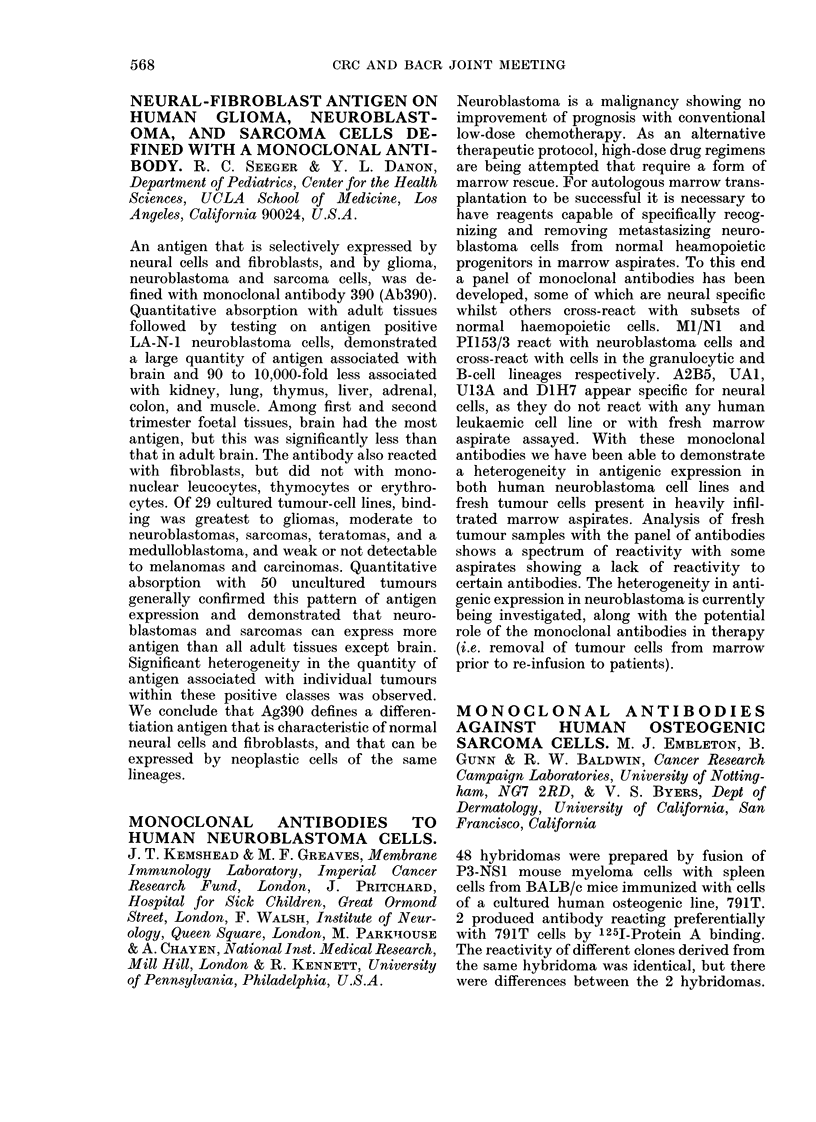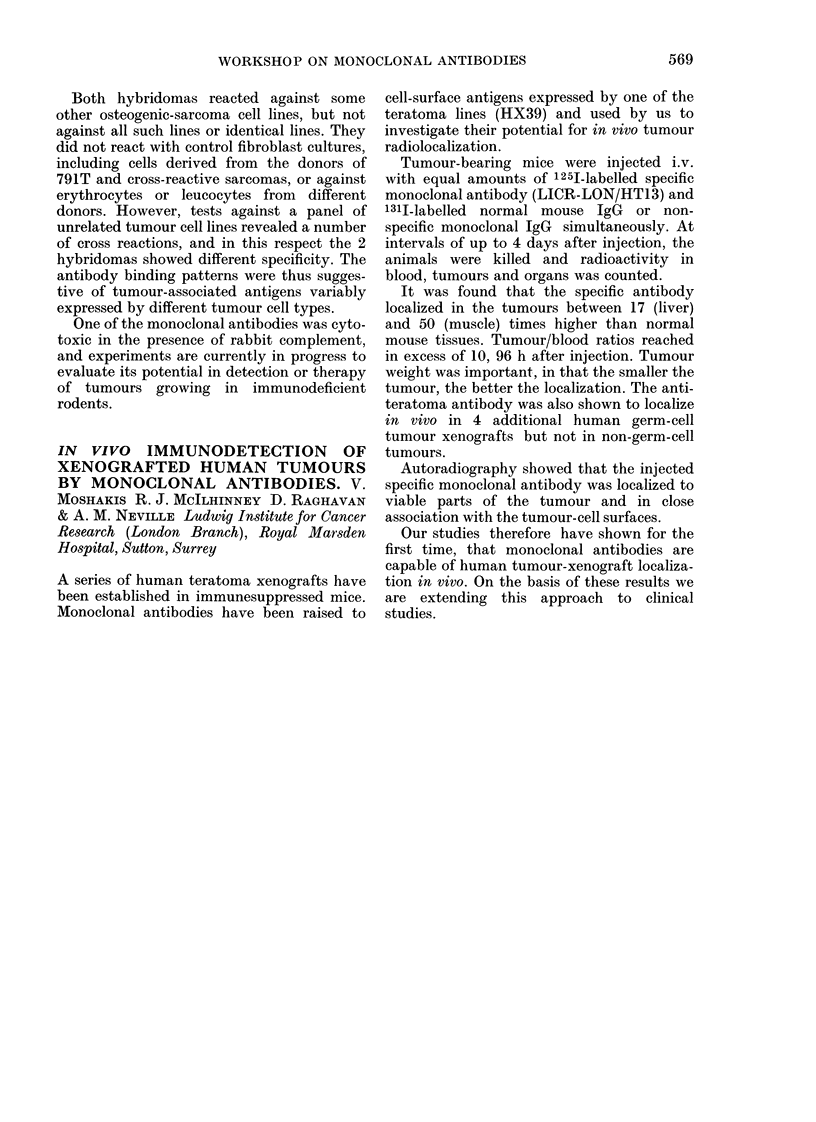# Proceedings of an international workshop on monoclonal antibodies to tumour antigens.

**DOI:** 10.1038/bjc.1981.82

**Published:** 1981-04

**Authors:** 


*Br. J. Cancer* (1981) **43**, 559

**CANCER RESEARCH CAMPAIGN**

IN CONJUNCTION WITH

**BRITISH ASSOCIATION FOR CANCER RESEARCH**

### PROCEEDINGS OF AN INTERNATIONAL WORKSHOP ON MONOCLONAL ANTIBODIES TO TUMOUR ANTIGENS

*Held at The Paterson Laboratories, Christie Hospital and Holt Radium Institute, Manchester M20 9BX, England*

7–9 December 1980

CONVENORS

M. MOORE, E. LENNOX AND M. J. EMBLETON

### WORKSHOP PARTICIPANTS

J. Arklie
K. D. Bagshawe
R. W. Baldwin
P. Beverley
W. Bodmer
J. Boyle
P. Brickell,
D. T. Brown
G. Brown
S. Carrell
M. Crumpton
W. Dippold
P. Edwards
M. J. Embleton
T. Feizi
C. Graham
M. F. Greaves
B. Gunn
V. Harrison
K . E. Hellström
T. Hince
K. James
G. Janossy
J. Johnson
J. T. Kemshead
R. H. Kennett
H. Koprowski
S. Kumar
L. G. Lajtha
E. Lennox
I. McConnell
J. O. D. McGee
A. J. McMichael
R. J. Marchmont
M. Moore
V. Moshakis
A. G. Munro
G. Riethmuller
R. Seeger
K. Sikora
J. Taylor-Papadimitriou
R. Tindle
V. van Heyningen
C. Vennegoor
H. Waldemann
R. Weiss,
Sir Michael Woodruff

**MONOCLONAL ANTIBODIES IN THE STUDY OF TUMOUR ANTIGENS.** H. KOPROWSKI, *The Wistar Institute, Philadelphia, PA* 19104, *U.S.A.*

Monoclonal antibodies (MA) were produced by hybridomas obtained through the fusion of mouse myeloma cells with lymphocytes obtained from the spleens of mice or rats immunized against a variety of human tumour antigens. Of the 11 MA that bind in radioimmunoassay (RIA) to human melanoma, 3 recognize the DR antigen, which is also expressed by human lymphocytes, 1 recognizes an antigen that is also expressed by some astrocytomas but not by any other cells and 6 define antigens expressed only by melanomas; 7 of these immunoprecipitate antigens of different mol. wts. Of 11 MA that bind to colorectal carcinoma (CRC), 1 defines carcinoembryonic antigen (180,000 mol. wt), 3 bind to a monosialoganglioside that can be isolated from CRC and meconium but not from other tissue, 3 react with neutral glycolipids extracted from CRC, and 2 immunoprecipitate a CRC antigen with mol. wt 21–28,000; 1 of these MA effectively suppresses the growth of CRC in nude mice. MA have also been raised through the immunization of animals with cells from astrocytoma, lung adenocarcinoma, lung oat-cell carcinoma and breast carcinoma; identification of the antigens recognized by these MA is still in preliminary stages.

**TWO ANTIGENS IDENTIFIED ON HUMAN MELANOMA CELLS BY MONOCLONAL ANTIBODIES:** K. E. HELLSTRÖM, I. HELLSTRÖM, J. P. BROWN, M-Y. YEH, R. G. WOODBURY & K. NISHIYAMA, *Fred Hutchinson Cancer Research Center, Seattle, Washington, U.S.A.*

We have immunized BALB/c mice with cultivated human melanoma cells and hybridized spleen cells from the immunized mice with NS-1 or Sp-2/0-Ag14 myeloma cells. The hybridomas have been screened by binding assays, and antibody specificities tested on a large panel of cells, including the immunizing melanoma, other melanomas, non-melanoma tumours of different types, B and T cell lines, and normal cells. Two antigens defined have been of particular interest. The first, Ag3.1, is expressed less by the melanoma, M1804, used for immunization, but can be detected in smaller amounts also on some other melanomas. An $IgG_{2a}$ antibody to 3.1 gives complement-dependent cytotoxicity as well as ADCC in the presence of human leucocytes. The antigen is expressed in tumour biopsy material. Antigen-negative variants can be selected from tumours expressing Ag3.1 and from Ag3.1$^+$ clones established from such tumours. The molecular nature of Ag3.1 is unknown; it is possibly a glycolipid.

The second, Agp97, is a glycoprotein of mol. wt 97,000 on SDS–PAGE. By using monoclonal antibodies to different antigenic determinant on the p97 molecule, a very sensitive "double determinant immunoassay" was developed, which was used to extensively analyse for Agp97 in various cells obtained from cultures, as well as from tumour and tissue biopsies. Using such assays we found that Agp97 is present on most tumours and, in small amounts, in most normal tissues, but certain melanomas have about 100 times more Agp97 than normal cells we have tested.

**PREPARATION OF MONOCLONAL ANTIBODIES AGAINST MYCOPLASMA.** C. VENNEGOOR*, A. POLAK† & A. JONKER*, **Netherlands Cancer Institute, Amsterdam, and †National Institute of Public Health, Bilthoven, The Netherlands*

Monoclonal antibodies were prepared against the human melanoma cell line SK-Mel-25. One million living SK-Mel-25 cells were injected into BALB/c mice i.p and s.c. Booster injections with $10^6$ living cells were given i.v. on Days 7, 14 and 35. Spleen cells were harvested 4 days after the last booster injection and fused with the myeloma cell line P3x 63 Ag8. The presence of anti-SK-Mel-25 antibodies in the supernatants was tested with $^{125}I$-$F(ab')_2$ anti-mouse IgG and IgM. Test/control ratios $>2$ were considered positive. One of the hybridomas was selected for subsequent cloning and search for specificity for melanoma. Positive reactions were found on a number of melanoma cell lines, but also on various control cell lines. In immunofluorescence tests patchy structures were visible that seemed to be attached very loosely to the cell surface. Closer examination gave indications that the monoclonal antibodies were directed against a mycoplasma that had infected the cell lines. There is now evidence that the monoclonal antibodies are directed against *Mycoplasma hyorhinis*.

These results demonstrate the necessity of thorough control on mycoplasma infections in cells used for preparation of monoclonal antibodies, since the SK-Mel-25 cells had been cultured in the presence of the antibiotic kanamycin, grew fast, and *did not contain readily detectable mycoplasma infection when used for immunization.*

### CELL-SURFACE ANTIGENS OF HUMAN MALIGNANT MELANOMAS DEFINED BY MONOCLONAL ANTIBODIES:

W. G. Dippold, K. O. Lloyd, L. Li, H. Ikeda, H. F. Oettgen & L. J. Old, *Memorial Sloan-Kettering Cancer Center, New York, NY* 10021

Eighteen mouse monoclonal antibodies were selected for reactivity with cell-surface antigens of the immunizing human melanoma cell line, SK-MEL-28. Six distinct antigenic systems were defined by direct serological assays and absorption tests, using a panel of 41 cell lines derived from normal and malignant human tissues. Biochemical analysis indicated that 2 of the antigens are glycoproteins with mol. wts of 95,000 and 150,000 (gp95 and gp150). Two other antigenic systems ($O_5$ and $R_{24}$ group) are associated with heat-stable molecules with the characteristics of glycoplipids. The remaining two antigens ($M_{19}$ and $R_8$) are heat labile, but molecular characterization has not been possible. Each of the antigenic systems has a distinctive pattern of distribution on various cell types, varying from a broad representation to more restricted occurrence. $O_5$ appears to be a species antigen, being present on virtually every human cell type tested. gp95, gp150, $M_{19}$ and $R_8$ are found on a characteristic proportion of melanomas, astrocytomas and epithelial cancers, as well as on normal kidney cells. The antigen defined by the $R_{24}$ antibody has the most restricted distribution of all; highest reactivity is found with melanomas and astrocytomas, whereas epithelial cell types, fibroblasts and cells of haemopoietic origin lack $R_{24}$. Although occurrence of gp95, gp150, $M_{19}$ and $R_8$ distinguishes a small subset of melanomas not expressing these antigens, $R_{24}$ is found on all melanoma cells.

### HUMAN MELANOMA-ASSOCIATED ANTIGEN(S) DETECTED BY MONOCLONAL ANTIBODIES.

S. Carrel, J.-P. Mach & R. S. Accolla, *Unit of Human Cancer Immunology, Lausanne Branch, Ludwig Institute for Cancer Research,* 1066 *Epalinges, Switzerland*

Hybridomas were derived from 5 fusions between mouse myeloma cells P3-NS1/1Ag4 or P3x63/Ag8 and spleen cells from mice immunized with membranes from 2 human melanoma cell lines Me43 and IGR-3. Of 193 hybrids obtained, 55 secreted antibodies that reacted with the melanoma cell lines used for immunization, as assayed by an indirect antibody-binding radioimmunoassay. After a first screening for the absence of reactivity on 2 non-melanoma control cell lines, a total of 9 hybrids, 3 from the first, 2 from the third, 3 from the fourth and 1 from the fifth fusion were selected and cloned by limiting dilution. The specificity of these monoclonal antibodies was then investigated with the same binding assay on a panel of cell lines derived from melanomas, non-melanoma tumours and normal peripheral-blood lymphocytes, normal skin fibroblasts and normal spermatozoa. After this specificity analysis, the 9 selected hybridoma products could be classified into 3 groups, antibodies binding to melanomas only, antibodies binding to melanomas and glioblastomas, and antibodies directed against HLA-DR antigens. By complement-dependent cytotoxicity experiments using $^{51}Cr$-labelled target cells, it was shown that antibodies from 3 clones were able to lyse melanoma target cells. Reciprocal binding inhibition tests using $^{3}H$-leucine-labelled antibodies showed that 3 hybridoma products cross-reacting with glioblastomas were directed against different antigenic determinants located on separated molecules.

### BIOCHEMICAL ANALYSIS OF MELANOMA-ASSOCIATED ANTIGENS DEFINED BY MONOCLONAL ANTIBODIES:

J. Johnson, G. Riethmüller & T. Meo, *Institut für Immunologie der Universität München, München, W. Germany*

Monoclonal antibodies were produced by immunizing mice with melanoma cell lines derived from living patients. Based on their reactivity with a cell panel consisting of cell lines of different tissue origin, the antibodies obtained were used to define 4 broad classes of melanoma-associated antigens. Sepharose-coupled monoclonal antibodies were used to isolate selected antigens from cell lysates of biosynthetically or surface-labelled cells and the antigens were analysed by SDS polyacrylamide gel electrophoresis and autoradiography.

Two antigens which are found on most tested melanoma and carcinoma cell lines but are absent from lymphoid lines have been analysed in detail. Antibody 15.75 precipitates a glycoprotein of 74 kilodaltons (kd) from the surface of both melanoma and carcinoma cell lines. A surface glycoprotein of 49 kd is precipitated by antibody 15.95 from melanoma and carcinoma cell lines. Both antigens can be biosynthetically labelled with $^{35}$S-methionine. GP49 is present on a teratocarcinoma cell line, and also on 3 foetal fibroblast lines which have been tested. Antibody 16.23 recognizes an antigen which was found only on the immunizing melanoma and on a lymphoblastoid cell line derived from the tumour donor. From the melanoma, this antibody precipitates 3 polypeptides of mol. wt 28, 31 and 38 kd. The antigen defined by antibody 16.54 is found on lymphoblastoid cell lines as well as on melanomas, though it is not detected in PBL or fibroblasts. From the surface of melanoma cells, this antibody precipitates 2 polypeptide chains of 95 and 18 kd. The genetic control of expression of these antigens is currently under investigation, using a series of interspecific somatic-cell hybrids containing various human chromosomes.

### MONOCLONAL ANTI-CEA ANTIBODIES: G. T. ROGERS, G. A. RAWLINS & K. D. BAGSHAWE, *Department of Medical Oncology, Charing Cross Hospital, London, W.6*

Somatic-cell hybridization and clonal-selection experiments have been used to produce 2 distinct monoclonal antisera against CEA. MS/1 which binds a maximum of 27% of radiolabelled CEA can be used at a dilution of 1 : 1000 for double-antibody RIA. Competitive binding with MS/1 has shown that 8600 ng/ml of unlabelled CEA extracted from tumour is required to produce 50% "displacement" of bound label. In contrast, only 30 ng/ml of CEA extracted from patients' sera is required to produce this displacement. Clinical RIA using MS/1 has shown MS/1-binding CEA to be associated with many forms of cancer, although its expression in the serum often differs from that of conventional CEA. The other monoclonal antibody, MA/200, is able to bind CEA more readily, with a maximum binding of 51% of 1 ng of [$^{125}$I]-CEA. Optimal double-antibody RIA conditions, obtained using MA/200 at a dilution of 1:20,000, produced 16% binding of radiolabelled CEA. Competitive-inhibition experiments with MA/200 have shown that, unlike MS/1, only 80 ng/ml of unlabelled tumour CEA is required to produce 50% displacement of bound label. Clinical RIA data obtained with MS/1 and MA/200 are being evaluated.

### ANTI-COLON-CARCINOMA ANTIBODIES ANALYSED WITH IMMUNOFLUORESCENCE IN FROZEN SECTIONS: J. BERRY, F. TAKEI & E. LENNOX, *MRC Laboratory of Molecular Biology, Hills Road, Cambridge*

Fourteen rat monoclonal antibodies raised against membranes from human colonic adenocarcinoma, showing *in vitro* binding to membranes, were assessed by indirect immunofluorescence on cryostat sections of fresh human tissue.

Seven supernates showed strong specific fluorescence against such components of normal human colonic mucosa as smooth muscle, goblet-cell mucin and epithelium. There were several different patterns of epithelial fluorescence, which depended on the antibody and the site from which the sample of mucosa was taken. None of the antibodies gave the same pattern, whilst all were consistent.

Activity on the corresponding colonic carcinoma was generally less than that on normal mucosa, or absent. One supernate showed enhanced antitumour activity and was also active against a crude extract of CEA. No supernate gave fluorescence with tumour and was inactive on normal colon.

Immunofluorescence and other histochemical techniques are available for separating different antibody activites against complex tissue antigens, and will help define the possible application of these antibodies.

### MONOCLONAL ANTIBODIES TO HUMAN CARCINOMA CELL LINES:

J. ARKLIE & W. F. BODMER, *Imperial Cancer Research Fund, London*

The following monoclonal antibodies have been raised by immunizing BALB/c mice with whole cells of carcinoma cell lines: antibodies 72B1 and AAP1 against D98/AH-2 (cervical carcinoma) and antibodies AUA1, D20L and C15L against LoVo (colonic carcinoma). AAP1 has been characterized as specific for the enzyme intestinal alkaline phosphatase that is produced ectopically by D98/AH-2. Characterization of the other antibodies by [$^{125}$I]-RAM trace binding assay against a panel of carcinoma, fibroblast and lymphoid cell lines, has shown that all are epithelium-specific, but that there are patterns of binding within the epithelial panel such that 72B1 is D98/AH-2 specific, D20L reacts with 4/14 lines, AUA1 with 13/14 lines (including 3 colon-carcinoma lines) and C15L with 12/14 lines. The mol. wts of the antigens recognized by C15L and D20L, as revealed by SDS–PAGE, were 60,000 and 40,000 respectively. In immunoperoxidase studies on formalin-fixed paraffin sections of normal tissue, D20L bound only to small intestine whereas AUA1 bound to both small and large intestine. Using the immunoperoxidase technique we are currently screening these monoclonal antibodies against a panel of tumour types.

### HUMAN MONOCLONAL ANTIBODIES TO LUNG-CANCER ANTIGENS.

K. SIKORA & R. WRIGHT, *MRC Clinical Oncology and Radiotherapeutics Unit, The Medical School, Hills Road, Cambridge*

Lymphocytes obtained from hilar and bronchial lymph nodes from 23 patients undergoing radical surgery for carcinoma of the bronchus were fused with established rat or mouse myeloma lines, using polyethylene glycol. Hybrids were obtained from 12 patients. There was no apparent correlation between successful fusion and the age, sex or blood group of the patient. 62% of the resultant hybrids were found to be secreting human immunoglobulin, by a sensitive protein A-coupled SRBC-lysis assay. Immunoglobulins synthesized by such hybrids were internally labelled with $^{3}$H-lysine, and their antibody activity against a variety of membrane preparations determined. Nine human monoclonal antibodies were found which bound to molecules on lung-cancer membranes but not to those found in normal lung membranes from the same patient. The determinants against which these antibodies were directed were found on lung tumours from several different patients.

The abstract which occupied this space was withdrawn at the last minute.

The abstract which occupied this space was withdrawn at the last minute.

**DISTRIBUTION OF HLA CLASS 1 ANTIGENS IN NORMAL HUMAN TISSUES AND MAMMARY CANCER.** J. O'D. McGee, K. A. Fleming, A. McMichael, J. Woods & J. Morton, *University of Oxford, Nuffield Departments of Pathology and Medicine, John Radcliffe Hospital, Oxford*

HLA antigens are glycoproteins which are presumed to be located exclusively on cell surfaces. These antigens are of two classes: Class 1 (HLA-A,B,C) and Class 2 (HLA-DR). It has been assumed from absorption experiments of HLA antisera with whole-tissue homogenates that these antigens are present in virtually all nucleated cells.

HLA antigens modulate some cell-mediated immune responses by major histocompatibility complex (MHC) restriction of T-cell-mediated immunity. This is of proven relevance in immunity to viral infections, and may also be relevant to T-cell-mediated responses to tumour cells.

In this paper, using monoclonal antibodies it is shown that HLA Class 2 antigens are expressed only on normal lymphoid cells, vascular endothelial, and certain types of epithelial cell (including mammary-duct epithelium). In contrast HLA (A,B,C) antigens are expressed in half of 19 human mammary cancers examined to date. A prospective study is underway to determine whether the expression of HLA Class 1 antigens on human mammary cancer cells is of prognostic significance since the natural history of these tumours is variable.

**APPLICATION OF MONOCLONAL ANTIBODY AGAINST A TUMOUR-SPECIFIC ANTIGEN ON A NATURALLY ARISING RAT MAMMARY CARCINOMA.** R. W. Baldwin, M. J. Embleton, B. Gunn, J. A. Jones, J. G. Middle, M. V. Pimm & M. R. Price, *Cancer Research Campaign Laboratories, University of Nottingham, Nottingham NG7 2RD*

Inter-specific hybridization of spleen cells from a WAB/Not rat immunized against syngeneic grafts of a naturally arising mammary carcinoma (Sp4) and P3-NS1-Ag4 mouse myeloma cells has been used to produce hybridomas secreting antibody which detects a tumour-specific antigen. The antibody secreted by one cloned cell line (Sp4 MoAb) has been used in a number of studies on the immunobiology of this rat mammary carcinoma. This includes tumour-antigen isolation using affinity chromatography of tumour extracts on Sp4 MoAb cross-linked on Sepharose 4B. Sp4 MoAb has also been evaluated as a drug carrier for therapy and for tumour localization using $^{125}$I-labelled antibody.

**A LIBRARY OF MONOCLONAL ANTIBODIES AGAINST HUMAN HAEMOPOIETIC CELLS: ANALYSIS OF ANTIGEN EXPRESSION DURING EARLY HAEMOPOIETIC DIFFERENTIATION AND APPLICATION TO THE DIFFERENTIAL DIAGNOSIS OF LEUKAEMIA.** M. F. Greaves, J. Robinson, R. Sutherland & R. A. Newman, *Membrane Immunology Laboratory, Imperial Cancer Research Fund, London*, G. Goldstein & P. Kung, *Ortho Pharmaceutical Corporation, Raritan, New Jersey, U.S.A.*, P. Edwards, *Ludwig Institute for Cancer Research, Sutton, Surrey* & A. McMichael, *Dept Surgery, Radcliffe Infirmary, Oxford*

A panel of 25 monoclonal antibodies with selective reactivity against one or more of each of the haemopoietic lineages (T, B lymphoid, erythroid, granulocytic, monocytic and platelet) in addition to a series of monoclonals specific for HLA-ABC, HLA-DR or $\beta_2$-microglobulin, have been assessed for their reactivity against a large series of leukaemias and leukaemic cell lines. Several important new observations emerged from this study including the remarkable heterogeneity of phenotype in T-cell malignancy (which may nevertheless parallel normal T-cell differentiation) and the detection of cryptic or equivocal erythroid or megakaryoblastic leukaemias with anti-glycophorin or anti-platelet antibodies respectively. Several antibodies which were independently produced react concordantly and may bind to the same structure; *e.g.* (T101, L17F12 and OKT1) (OKT6 and NA134) (J-5 anti-cALL and rabbit anti-cALL). A number of the OKT series of anti-T monoclonals have interesting features; *e.g.* OKT11 binds to E-rosette receptors, and OKT9 binds to a structure with a widespread expression on tumour cells. The biochemical features of some of these "leukaemia-associated" antigens identified by these monoclonals have been investigated; *e.g.* J-5 cALL (=gp100), BA-2 anti-ALL (=p26), OKT9 (=p95/100), Ia/HLA-DR ($=p28 \pm p30 + p33$).

We are attempting to compile a phenotypic profile of normal haemopoietic differentiation using these monoclonals, and have been able to delineate a sequence of cell-surface antigen expression during erythroid differentiation from the BFU-E to the mature erythrocyte, by fluorescence-activated cell sorting. This type of analysis provides some interesting insight into the timing of appearance of particular structures of interest; *e.g.* HLA-DR or BFU-E, glycophorin on the post-CFU-E cells, Band III on erythrocytes.

### THE USE OF MONOCLONAL ANTIBODIES IN HISTOLOGICAL ANALYSIS AND MARROW TRANSPLANTATION: A REVIEW.

G. Janossy, *Department of Immunology, Royal Free Hospital, London, NW3*

The lecture reviews three areas. First, a few examples of markers for normal differentiation antigens which can be used to find malignant cells. A typical example is the identification of cells expressing thymocyte phenotype in marrow. These may correspond to thymic ALL blasts. Similarly TdT+ cells in the testis and the cerebrospinal fluid indicate the presence of leukaemia.

Secondly, the current data on identifying lymphocyte subpopulations by monoclonal antibodies in tissue sections. As monoclonal antibodies to well defined functional lymphoid subsets exist, this technology may provide a much needed link between classical histology and lymphocyte functional assays carried out *in vitro*. It is very likely that the analysis of the reactivity pattern of monoclonal antibodies on tissue sections will provide new reagents against monocyte–macrophage and B-lymphocyte subpopulations.

Thirdly, a few simple techniques standardize monoclonal antibodies for marrow transplantation. These include the morphological analysis of bone marrow precursor cells (such as TdT+ putative lymphoid precursors and myeloblasts) and functional assays (*e.g.* CFU-C test). These tests were used to analyse a large panel of reagents. OKT3 antibody, made by Ortho Laboratories, seems to be the best candidate for attempting to remove T lymphocytes in order to prevent graft-versus-host disease in allogeneic marrow transplantation. This antibody has opsonizing capacity. The clinical results with this antibody in GVH prevention (3 patients transplanted at the Royal Free Hospital; G. Prentice, personal communication) are encouraging. The antibody did not have harmful side effects, but longer follow-up and more experience is necessary before final conclusions can be drawn.

### MONOCLONAL ANTIBODIES TO HUMAN CELL SURFACE ANTIGENS.

G. Brown & I. C. M. MacLennan, *Dept of Immunology, University of Birmingham*, J. M. Bastin, *Nuffield Dept of Clinical Medicine, University of Oxford* & F. M. Kourilsky, *Centre d'Immunologie, Marseille*

Mice were immunized with cells from patients with acute myeloblastic leukaemia and chronic lymphocytic leukaemia. These were presented as membrane preparations, whole cells and homogenized cells. The hybrid clones, selected by indirect radioactive binding against the immunizing cells, produced antibody against: (1) antigens which are found on a wide range of normal cells (25/39); (2) leucocyte-associated antigens (13/39) and (3) a lymphoid-associated antigen (1/39). Primary selection of hybridoma cultures was also based on the production of mouse immunoglobulin. Forty-seven immunoglobulin producing hybridomas were selected, of which only 20 had activity when tested using an indirect radioactive binding assay against the immunizing cells. Detailed analysis of 13 of the remaining 27 has revealed antibodies reactive against: (1) an antigen expressed in relatively low amounts on myeloid cells; (2) an antigen expressed on cell lines tested, large mononuclear cells and proliferating cells in squamous epithelium and germinal centres; (3) a subpopulation (1–6%) of peripheral-blood lymphocytes and (4) cytoplasmic components. It is concluded that primary selection of hybridomas on the basis of mouse immunoglobulin production is an important adjunct to selection using surface-binding assays against the immunizing cells.

**ANTIGENS ON THE SURFACE OF THE BURKITT LYMPHOMA LINE DAUDI.** V. van Heyningen, K. Guy, D. Crichton, M. Moxley & C. M. Steel, *MRC Clinical and Population Cytogenetics Unit, Western Royal Hospital, Edinburgh EH*4 2X*U*

We have raised 4 B-cell-specific monoclonal antibodies (MCAs) following immunization of BALB/c mice with DAUDI cells. Three of the MCAs, DA6-231, -164 and -147, define non-polymorphic determinants. The fourth, DA6-127, is directed against a polymorphic antigen. Preliminary biochemical analysis suggests that 231, 164 and 147 each precipitate HLA-DR-like 33K/28K dimeric molecules. Binding of 164 on a given cell line or on peripheral B cells is always lower than of 231. 164 partially inhibits the binding of 231, whilst 231 completely inhibits 164. 164 has also been found absent from a $231^+$ leukaemic marrow sample. We consider that 164 defines a subset of 231-determinant-bearing molecules and may identify the product of a second DR-like locus. 147 is very weakly represented on peripheral B lymphocytes but is strongly present on all B lymphoblastoid lines and on all $231^+$ leukaemic cells tested (including several chronic lymphatic and a myelomonocytic) as well as on a B lymphoma and an established myeloma line. 164 and 147 strongly inhibit mixed lymphocyte reactions in the absence of complement; 231 inhibits moderately under the same conditions.

**MONOCLONAL ANTIBODIES RAISED AGAINST HUMAN ALPHAFOETO-PROTEIN (AFP).** V. van Heyningen*, D. Crichton*, S. Lawrie*, L. Barron† & D. J. H. Brock†, **MRC Clinical and Population Cytogenetics Unit and* †*Department of Human Genetics, Western General Hospital, Edinburgh EH*4 2X*U*

Fusion of spleen cells, from BALB/c mice immunized with purified human AFP, with the $X63NS_1$ myeloma line, has led to the isolation of 5 anti-AFP monoclonal antibody (MCA) secreting clones. Three of these have been extensively studied. 21.1 (IgA), 21.2 ($IgG_1$) and 22.4 ($IgG_1$) all bind [$^{125}$I]-AFP in a polyethylene glycol (PEG) dependent precipitation assay. However, the system is much more sensitive to carrier-protein concentration than when conventional antisera are used. Co-operativity is found when combinations of 2 or all 3 MCAs are used to generate antibody dilution or radioimmunoassay (RIA) curves. 21.2-derived ascites fluid alone or in combination with the other MCAs can be used at $10^{-4}$ dilution in RIA. A solid-phase method using Sepharose-bound second antibody gives precipitation levels comparable with the PEG system. $^{125}$I-labelled 21.2 performs very well in an immunoradiometric assay (IRMA) with conventional sheep anti-AFP used as the solid phase. (IRMA work carried out by W. H. Hunter *et al.*, MRC Immunoassay Team.)

**MONOCLONAL ANTIBODIES TO HUMAN LYMPHOMA CELLS.** P. Brickell, I. McConnell & C. Milstein, *MRC Centre, Hills Road, Cambridge, CB*2 2*QH, England*

A number of monoclonal antibodies to EBV-transformed B cells have been raised in rats. Some of these monoclonal antibodies were shown to be lymphoma-cell specific, in that they only reacted with *in vitro* cultured cell lines derived from patients with Burkitt's lymphoma. These same monoclonal antibodies failed to react with EBV-transformed lymphoblastoid cell lines and with normal lymphocytes. The lymphoma-specific monoclonal antibodies did not distinguish between $EBV^-$ and $EBV^+$ lymphoma lines.

A monoclonal antibody to the HLA-DR locus was also described. The determinant recognized by this antibody is monomorphic in man but polymorphic in mice. Mapping studies with inbred recombinant strains of mice show that this antigen is associated with H-2 haplotypes B, D and Q, and is coded for by genes within the IA sub-region. This monoclonal antibody was found to precipitate 3 polypeptide chains from surface-labelled Raji cells. These included the expected 34,000 dalton and 28,000 dalton polypeptides, as well as a third component of 25,000 dalton. The origin of the latter polypeptide is not known. It may represent a post-translational modification or a non-glycosylated form of the 28,000 dalton chain. Alternatively, this monoclonal antibody may be precipitating two distinct species of $\beta$ chain.

**MONOCLONAL AUTOANTIBODIES WITH BLOOD GROUP I AND i SPECIFICITIES.** T. Feizi, *Clinical Research Centre, Watford Road, Harrow, Middlesex, HA1 3UJ*

Among the erythrocyte autoantibodies in chronic cold-agglutinin disease, the majority are monoclonal IgM proteins directed against the blood group I and i antigens. The i antigen is a linear and I a branched carbohydrate chain made up of N-acetyllactosamine (Gal$\beta$1→4GlcNAc) units as follows:

i Gal$\beta$1→4GlcNAc$\beta$1→3Gal$\beta$1
↓
4GlcNAc$\beta$1→3Gal$\beta$1—

Gal$\beta$1→4GlcNAc$\beta$1
↘
I $^{6}_{3}$Gal$\beta$1→4GlcNAc$\beta$1
↗ ↓
Gal$\beta$1→4GlcNAc$\beta$1 3Gal$\beta$1—

These structures occur on a variety of cell types and are carried on glycoproteins and glycosphingolipids. They can be further glycosylated and converted into blood-group ABH-active structures and gangliosides. Their antigenicities are totally or partially masked by these additional glycosylations.

The monoclonal anti-I and anti-i antibodies of individual donors recognize different oligosaccharide domains on these structures (Feizi *et al.* (1979) *J. Exp. Med.*, **149**, 975) and they are proving valuable reagents in studies of antigenic changes associated with (a) ontogenesis of erythroid cells, (b) differentiation of teratocarcinomas *in vitro* and (c) diseases of the gastric mucosa.

**MONOCLONAL ANTIBODIES AGAINST HUMAN NEUROBLASTOMA ANTIGENS AND HUMAN LEUKEMIA ANTIGENS.** R. H. Kennett, Z. Jonak, B. Haas & R. Byrd, *Department of Human Genetics, Univ. of Pa. School of Medicine, Philadelphia, Pa.* 19104, *U.S.A.*

Monoclonal antibodies were made against human neuroblastoma cells precoated with an antiserum against a human lymphoblastoid line. The antibody PI153/3 reacts with human tumours of neuroectodermal origin and with foetal brain. The antigen is the carbohydrate portion of a cell-surface glycoprotein. A cross-reacting antigen is present on B-cell and null-cell leukaemias, and on immature lymphocytes. A second antibody, P3B1C3, reacts with B cells and null cells, and used in combination these antibodies makes it possible to identify neuroblastomas in marrow, and also to identify and classify leukaemia cells.

We have also prepared a panel of monoclonal antibodies against Reh, an acute lymphoblastic leukaemia (ALL) cell line derived from a patient with non-B non-T ALL. Monoclonal antibodies obtained after immunization with whole cells, KCl glycoprotein extracts, solubilized vesicle preparations, or cell-culture supernatants, were compared with regard to number of positive hybrid clones and specificity for Reh. Immunization with concentrated culture supernatants produced more antibodies that, after screening on 4 cell types, showed specificity for Reh.

A model in which monoclonal antibodies may react with molecules on the cell surface which are involved in growth control is presented. It is suggested that these antibodies may mimic the effect of the normal growth-control messenger and stimulate the Reh cells to stop dividing and differentiate. Four of the antibodies made against Reh have been shown to inhibit the uptake of [$^{3}$H]-dT. Three of these also inhibit the proliferation of the T-cell line CCRF/CEM. The fourth does not inhibit CCRF/CEM. It is suggested that monoclonal antibodies will be useful for identifying all surface molecules involved in growth control and differentiation mechanisms.

**NEURAL-FIBROBLAST ANTIGEN ON HUMAN GLIOMA, NEUROBLASTOMA, AND SARCOMA CELLS DEFINED WITH A MONOCLONAL ANTIBODY.** R. C. SEEGER & Y. L. DANON, *Department of Pediatrics, Center for the Health Sciences, UCLA School of Medicine, Los Angeles, California* 90024, *U.S.A.*

An antigen that is selectively expressed by neural cells and fibroblasts, and by glioma, neuroblastoma and sarcoma cells, was defined with monoclonal antibody 390 (Ab390). Quantitative absorption with adult tissues followed by testing on antigen positive LA-N-1 neuroblastoma cells, demonstrated a large quantity of antigen associated with brain and 90 to 10,000-fold less associated with kidney, lung, thymus, liver, adrenal, colon, and muscle. Among first and second trimester foetal tissues, brain had the most antigen, but this was significantly less than that in adult brain. The antibody also reacted with fibroblasts, but did not with mononuclear leucocytes, thymocytes or erythrocytes. Of 29 cultured tumour-cell lines, binding was greatest to gliomas, moderate to neuroblastomas, sarcomas, teratomas, and a medulloblastoma, and weak or not detectable to melanomas and carcinomas. Quantitative absorption with 50 uncultured tumours generally confirmed this pattern of antigen expression and demonstrated that neuroblastomas and sarcomas can express more antigen than all adult tissues except brain. Significant heterogeneity in the quantity of antigen associated with individual tumours within these positive classes was observed. We conclude that Ag390 defines a differentiation antigen that is characteristic of normal neural cells and fibroblasts, and that can be expressed by neoplastic cells of the same lineages.

**MONOCLONAL ANTIBODIES TO HUMAN NEUROBLASTOMA CELLS.** J. T. KEMSHEAD & M. F. GREAVES, *Membrane Immunology Laboratory, Imperial Cancer Research Fund, London*, J. PRITCHARD, *Hospital for Sick Children, Great Ormond Street, London*, F. WALSH, *Institute of Neurology, Queen Square, London*, M. PARKHOUSE & A. CHAYEN, *National Inst. Medical Research, Mill Hill, London* & R. KENNETT, *University of Pennsylvania, Philadelphia, U.S.A.*

Neuroblastoma is a malignancy showing no improvement of prognosis with conventional low-dose chemotherapy. As an alternative therapeutic protocol, high-dose drug regimens are being attempted that require a form of marrow rescue. For autologous marrow transplantation to be successful it is necessary to have reagents capable of specifically recognizing and removing metastasizing neuroblastoma cells from normal heamopoietic progenitors in marrow aspirates. To this end a panel of monoclonal antibodies has been developed, some of which are neural specific whilst others cross-react with subsets of normal haemopoietic cells. M1/N1 and PI153/3 react with neuroblastoma cells and cross-react with cells in the granulocytic and B-cell lineages respectively. A2B5, UA1, U13A and D1H7 appear specific for neural cells, as they do not react with any human leukaemic cell line or with fresh marrow aspirate assayed. With these monoclonal antibodies we have been able to demonstrate a heterogeneity in antigenic expression in both human neuroblastoma cell lines and fresh tumour cells present in heavily infiltrated marrow aspirates. Analysis of fresh tumour samples with the panel of antibodies shows a spectrum of reactivity with some aspirates showing a lack of reactivity to certain antibodies. The heterogeneity in antigenic expression in neuroblastoma is currently being investigated, along with the potential role of the monoclonal antibodies in therapy (*i.e.* removal of tumour cells from marrow prior to re-infusion to patients).

**MONOCLONAL ANTIBODIES AGAINST HUMAN OSTEOGENIC SARCOMA CELLS.** M. J. EMBLETON, B. GUNN & R. W. BALDWIN, *Cancer Research Campaign Laboratories, University of Nottingham, NG7 2RD*, & V. S. BYERS, *Dept of Dermatology, University of California, San Francisco, California*

48 hybridomas were prepared by fusion of P3-NS1 mouse myeloma cells with spleen cells from BALB/c mice immunized with cells of a cultured human osteogenic line, 791T. 2 produced antibody reacting preferentially with 791T cells by $^{125}$I-Protein A binding. The reactivity of different clones derived from the same hybridoma was identical, but there were differences between the 2 hybridomas.

Both hybridomas reacted against some other osteogenic-sarcoma cell lines, but not against all such lines or identical lines. They did not react with control fibroblast cultures, including cells derived from the donors of 791T and cross-reactive sarcomas, or against erythrocytes or leucocytes from different donors. However, tests against a panel of unrelated tumour cell lines revealed a number of cross reactions, and in this respect the 2 hybridomas showed different specificity. The antibody binding patterns were thus suggestive of tumour-associated antigens variably expressed by different tumour cell types.

One of the monoclonal antibodies was cytotoxic in the presence of rabbit complement, and experiments are currently in progress to evaluate its potential in detection or therapy of tumours growing in immunodeficient rodents.

### *IN VIVO* IMMUNODETECTION OF XENOGRAFTED HUMAN TUMOURS BY MONOCLONAL ANTIBODIES.

V. Moshakis R. J. McIlhinney D. Raghavan & A. M. Neville *Ludwig Institute for Cancer Research (London Branch), Royal Marsden Hospital, Sutton, Surrey*

A series of human teratoma xenografts have been established in immunesuppressed mice. Monoclonal antibodies have been raised to cell-surface antigens expressed by one of the teratoma lines (HX39) and used by us to investigate their potential for *in vivo* tumour radiolocalization.

Tumour-bearing mice were injected i.v. with equal amounts of $^{125}$I-labelled specific monoclonal antibody (LICR-LON/HT13) and $^{131}$I-labelled normal mouse IgG or non-specific monoclonal IgG simultaneously. At intervals of up to 4 days after injection, the animals were killed and radioactivity in blood, tumours and organs was counted.

It was found that the specific antibody localized in the tumours between 17 (liver) and 50 (muscle) times higher than normal mouse tissues. Tumour/blood ratios reached in excess of 10, 96 h after injection. Tumour weight was important, in that the smaller the tumour, the better the localization. The anti-teratoma antibody was also shown to localize *in vivo* in 4 additional human germ-cell tumour xenografts but not in non-germ-cell tumours.

Autoradiography showed that the injected specific monoclonal antibody was localized to viable parts of the tumour and in close association with the tumour-cell surfaces.

Our studies therefore have shown for the first time, that monoclonal antibodies are capable of human tumour-xenograft localization *in vivo*. On the basis of these results we are extending this approach to clinical studies.